# A Genome-Wide Screen in Mice To Identify Cell-Extrinsic Regulators of Pulmonary Metastatic Colonisation

**DOI:** 10.1534/g3.120.401128

**Published:** 2020-04-03

**Authors:** Louise van der Weyden, Agnieszka Swiatkowska, Vivek Iyer, Anneliese O. Speak, David J. Adams

**Affiliations:** Wellcome Sanger Institute, Wellcome Genome Campus, Hinxton, Cambridge, CB10 1SA, United Kingdom

**Keywords:** metastasis, metastatic colonisation, microenvironment, B16-F10, lung, mutant, mouse

## Abstract

Metastatic colonization, whereby a disseminated tumor cell is able to survive and proliferate at a secondary site, involves both tumor cell-intrinsic and -extrinsic factors. To identify tumor cell-extrinsic (microenvironmental) factors that regulate the ability of metastatic tumor cells to effectively colonize a tissue, we performed a genome-wide screen utilizing the experimental metastasis assay on mutant mice. Mutant and wildtype (control) mice were tail vein-dosed with murine metastatic melanoma B16-F10 cells and 10 days later the number of pulmonary metastatic colonies were counted. Of the 1,300 genes/genetic locations (1,344 alleles) assessed in the screen 34 genes were determined to significantly regulate pulmonary metastatic colonization (15 increased and 19 decreased; *P* < 0.005 and genotype effect <-55 or >+55). While several of these genes have known roles in immune system regulation (*Bach2*, *Cyba*, *Cybb*, *Cybc1*, *Id2*, *Igh-6*, *Irf1*, *Irf7*, *Ncf1*, *Ncf2*, *Ncf4* and *Pik3cg*) most are involved in a disparate range of biological processes, ranging from ubiquitination (*Herc1*) to diphthamide synthesis (*Dph6*) to Rho GTPase-activation (*Arhgap30* and *Fgd4*), with no previous reports of a role in the regulation of metastasis. Thus, we have identified numerous novel regulators of pulmonary metastatic colonization, which may represent potential therapeutic targets.

Metastasis is the spread of cancer cells to a secondary site within the body, and is the leading cause of death for cancer patients. This is a multi-step process that is initiated when the tumor cells breach the basement membrane, that separate them from the other tissue layers, and enter the circulatory or lymphatic system, where they are known as ‘circulating tumor cells’ (CTCs) and are able to travel all over the body. The CTCs must leave the circulation (‘extravasate’) at a distant site, where they are known as ‘disseminated tumor cells’ (DTCs). The DTCs need to overcome numerous barriers to survive in the ‘foreign’ environment and be able to proliferate (‘colonization’), before developing into overt metastases and becoming a clinical problem. CTCs can be found in the patient’s blood at the time of diagnosis of a primary tumor, and thus it is highly likely that the early stages of metastasis (the presence of DTCs) has already occurred in these patients, especially since surgical excision of a primary tumor does not always prevent metastasis (Talmadge and Fidler 2010). Indeed, studies have demonstrated that the early steps of the metastatic process are relatively efficient, with the post-extravasation regulation of tumor growth (‘colonization’) being critical in determining metastatic outcome (Chambers *et al.* 2001). Thus, the prevention of primary tumor cells from entering the circulation is unlikely to be of therapeutic benefit, and a focus on preventing the survival of the CTCs and/or subsequent growth of the DTCs would seem a more feasible approach (Fidler and Kripke 2015).

The survival and growth of metastatic cells involves contributions from both tumor cell- intrinsic factors and tumor cell- extrinsic factors such as the microenvironment (‘host’), which includes stromal cells and the immune system (Quail and Joyce 2013). In recent years there has been a revolution in our understanding of the role that host factors, such as the immune system, stroma and vasculature play in the process of cancer progression. This is evidenced by the development of agents, such as checkpoint inhibitors, that provoke the immune system to identify and eliminate cancer cells. Importantly, studies in mice have made a significant contribution to these breakthroughs, such as with the clinically relevant PD-1 (Zago *et al.* 2016) and CTLA4 receptors (Leach *et al.* 1996), which were first identified and functionally characterized using mouse model systems. For this reason we sort to develop a genetic screen to identify new genes as tumor cell- extrinsic regulators of metastatic colonization.

In designing our screen we aimed to, where possible, unbiasedly screen mouse mutants to identify new genes involved in colonization of the lung, a common site of metastatic seeding for many tumor types. To this end, we used the ‘experimental metastasis assay’, which we have previously demonstrated is a sensitive, robust, and high-throughput method for *in vivo* quantification of the ability of metastatic tumor cells to colonize a secondary organ (Speak *et al.* 2017), to screen mutant mouse lines generated as part of the International Mouse Phenotyping Consortium (Meehan *et al.* 2017). In this paper we describe a collection of mutants identified over 7 years of screening (1,344 mutant mouse lines). This study reveals previously unappreciated pathways and processes that regulate this biology.

## Materials & Methods

### Mice

The mutant mice were generated as part of the International Mouse Phenotyping Consortium (Meehan *et al.* 2017), using either targeted embryonic stem cell clones obtained from the European Conditional Mouse Mutagenesis (EUCOMM) Program/Knockout Mouse Project (KOMP)-CSD collection or EUCOMMTools or CRISPR/Cas9 technology to either genetrap the target transcript or disrupt a critical exon or to create a point mutation, as detailed previously (van der Weyden *et al.* 2017b). The vast majority of lines (>98%) were on the C57BL/6 background, with other strain backgrounds including 129 and FVB (strain-matched control mice were always used for each mutant line). The care and use of all mice in this study were in accordance with the Home Office guidelines of the UK and procedures were performed under a UK Home Office Project license (PPL 80/2562 or P6B8058B0), which was reviewed and approved by the Sanger Institute’s Animal Welfare and Ethical Review Body. All mice were housed in individually ventilated cages in a specific pathogen free environment. The diet, cage conditions and room conditions of the mice were as previously reported (van der Weyden *et al.* 2017a).

### Cells for tail vein injection

The B16-F10 mouse melanoma cell line was purchased from ATCC (CRL-6475), genetically validated, and maintained in DMEM with 10% (v/v) fetal calf serum and 2 mM glutamine, 100 U/mL penicillin/streptomycin at 37°, 5% CO_2_. The cell line was screened for the presence of mycoplasma and mouse pathogens (at Charles River Laboratories, USA) before culturing and never cultured for more than five passages.

### Experimental metastasis assay

B16-F10 (4-5 × 10^5^) cells resuspended in 0.1 mL phosphate buffered saline (PBS) were injected into the tail vein of 6- to 12-week-old sex-matched syngeneic control and mutant mice. After 10 (± 1) days the mice were humanely killed, their lungs removed and washed in PBS and the number of metastatic foci counted macroscopically. The use of the experimental assay as a screen for metastatic colonization ability has been previously described (Speak *et al.* 2017).

### Statistics and bioinformatic analysis

The raw data (number of metastatic foci counted in each mutant mouse relative to the wildtype controls) from each cohort of mice was subjected to the non-parametric Mann-Whitney *U*-test. An integrative data analysis (mega-analysis) was performed on the results from all mutant mouse lines that had been tested in ≥ 3 independent cohorts, and was completed using R (package nlme version 3.1) as previously described (van der Weyden *et al.* 2017b). Using the Mouse Genome Database Informatics (MGI) portal (http://www.informatics.jax.org, v6.14), all 1,300 mutant lines screened were separated into unique symbols and annotated with molecular function using the Gene Ontology (GO) chart tool (Bult *et al.* 2019) excluding annotations that were Inferred from Electronic Annotation (IEA). Phenotypic information (MP-to-genotype) was pulled from MGI using MouseMine (Motenko *et al.* 2015) and the phenotypes collapsed to the parental term of the mouse phenotype hierarchy.

### Data availability

Table S1 lists the targeted genetic regions that were mutated in the genetically modified mice used in the screened. Table S2 is the complete data set (number of metastatic colonies) for all the mice comprising the 1,344 alleles screened (consisting of 23,975 individual mice). Table S3 explains how to interpret the data for the screen supplied in Table S2. Supplemental material available at figshare: https://doi.org/10.25387/g3.11815296.

## Results

**Figure 1:**
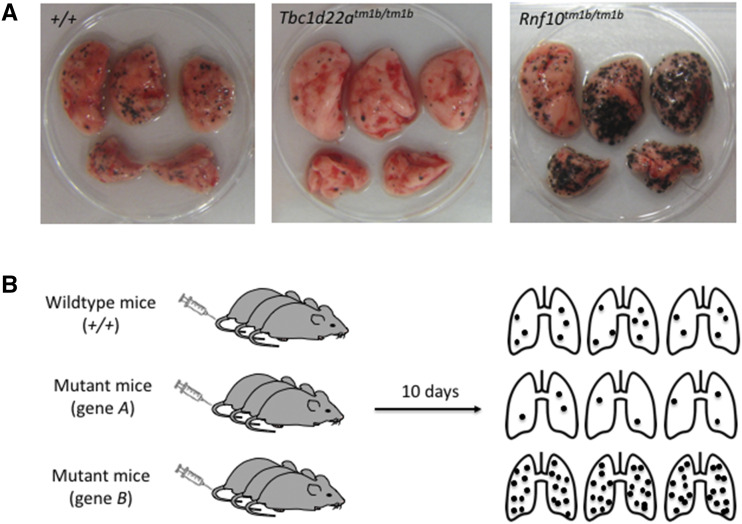
The metastatic colonization assay. (A) Representative macroscopic image of lungs from wildtype (+/+) and mutant (*Tbc1d22a^tm1b/tm1b^* and *Rnf10^tm1b/tm1b^*) mice 10 days after tail vein dosing with B16-F10 melanoma cells, demonstrating examples of decreased and increased metastatic colonization, respectively. (B) Schematic of the B16-F10 pulmonary metastasis screen, showing that a cohort of mice consists of wildtype mice and groups of different mutant mice, all of which are tail vein dosed with the B16-F10 melanoma cells, and then the number of pulmonary metastatic colonies counted 10 days later (the ‘metastatic ratio’ of a mutant line is derived by dividing the average of the metastases for a mutant group by the average number of metastases for the wildtype group).

Tail vein injection of mouse melanoma B16-F10 cells primarily results in pulmonary colonization (due to the capillary beds in the lungs being the first ones encountered by the cells in the arterial blood after leaving the heart). As these cells are pigmented (melanin granules) their colonization of the lungs can be determined by macroscopic counting of the number of black metastatic foci on the lungs (Figure 1A). Sex- and age-matched wildtype mice were concomitantly dosed with the cohorts of mutant mice (Figure 1B), and the results from mutant mice were only compared to the wildtype mice dosed at the same time (due to day-to-day variations in the assay, and factors such as sex and age of the mice affecting metastatic burden (Speak *et al.* 2017)). A ‘metastatic ratio’ (MR) was determined for each mutant mouse line, which was calculated as the average number of metastatic colonies for the mutant line relative to the average number of metastatic colonies for concomitantly dosed wildtype mice. If a mutant mouse line showed a MR of <0.6 or >1.4 (and Mann-Whitney *P* < 0.05), additional cohorts were assayed (n ≥ 2, assayed on independent days). An integrative data analysis (IDA) was performed on the whole dataset and those with *P* < 0.005 (Hochberg) and a biological effect (‘genotype effect’) of ≤ -55 or ≥ +55 were classified as ‘hits’. A biological filter was applied as we were only interested in determining robust (strong) regulators of metastatic colonization.

We used *Entpd1* and *Hsp90aa1* mutant mice as positive controls, as the literature suggested they should show altered metastatic burden. *Entpd1* (*ectonucleoside triphosphate diphosphohydrolase 1*) encodes the plasma membrane protein CD39. The enzymatic activity of CD39 (NTPDase I), together with CD73 (ecto-5′-nucleotidase), result in the phosphohydrolysis of extracellular ATP into adenosine, which acts as an immunosuppressive pathway through the activation of adenosine receptors (Stagg and Smyth 2010). *Entpd1*-deficient mice that were administered B16-F10 mouse melanoma cells and MC-38 mouse colon cancer cells via the hepatic portal vein (experimental metastasis assay) were found to develop significantly fewer hepatic metastases than wildtype (control) mice (Sun *et al.* 2010). In agreement with this, we found that *Entpd1* mutant mice showed significantly reduced numbers of pulmonary metastatic colonies after tail vein dosing with B16-F10 cells, relative to wildtype mice. *Hsp90aa1* (*heat shock protein 90 alpha family class A member 1*) encodes a molecular chaperone that functions to aid in the proper folding of specific target proteins (“clients”), including numerous kinases, transcription factors and steroid hormone receptors (Li *et al.* 2012). Systemic administration of a mitochondrial-targeted, small-molecule Hsp90 inhibitor (Gamitrinib) to Transgenic Adenocarcinoma of the Mouse Prostate (TRAMP) mice inhibited the formation of localized prostate tumors, as well as the spread of metastatic prostate cancer to abdominal lymph nodes and liver (Kang *et al.* 2011). In agreement with this, we found that *Hsp90aa1* mutant mice showed significantly reduced numbers of pulmonary metastatic colonies after tail vein dosing with B16-F10 cells, relative to wildtype mice. Both *Entpd1* and *Hsp90aa1* were classified as ‘hits’ using the integrated data analysis (IDA) approach, thus we were confident that our screening methodology was robust.

**Figure 2:**
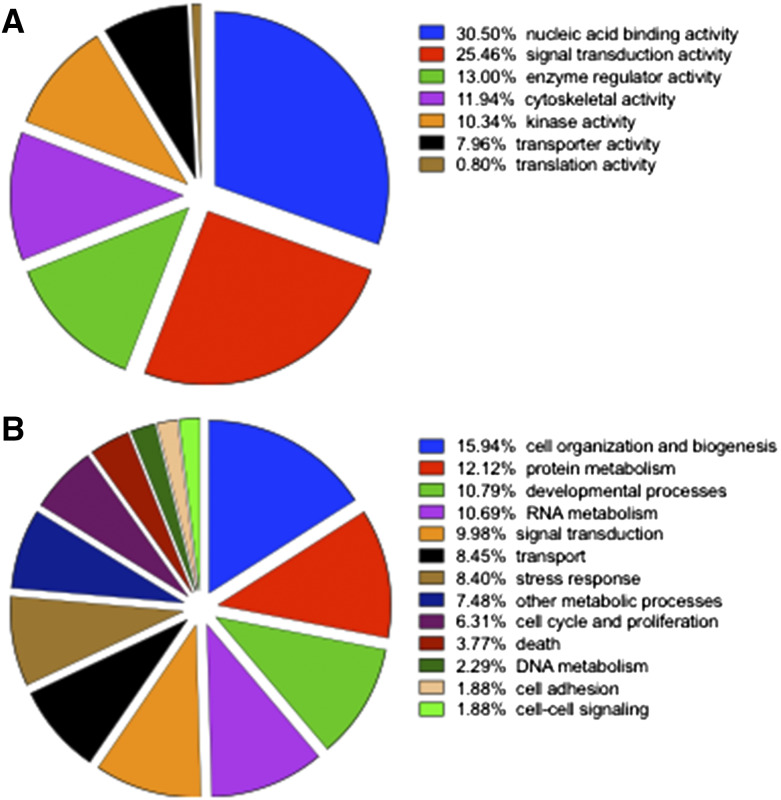
Gene Ontology annotation of the 1,300 mutant mouse lines screened as detailed in Methods. A) Molecular functions of genes screened and B) Biological processes of genes screened.

We have previously published the results of screening 810 mutant mouse lines and showed that endothelial SPNS2 can regulate metastatic colonization by sphingosine-1-phosphate (S1P)-mediated control of lymphocyte trafficking (van der Weyden *et al.* 2017a; van der Weyden *et al.* 2017b). Here we have included an additional 534 mutant mouse lines, to make a total of 1,344 mutant mouse lines (1,300 unique genes/genetic loci) screened. The mutant mouse lines were randomly selected and the genes/genetic loci (Table S1) cover a diverse range of molecular functions (Figure 2A) and are involved in many different biological processes (Figure 2B). Of the 1,300 mutant lines screened (representing unique genes/genetic loci), 1,247 lines (96%) carried alleles that targeted single protein coding genes, with the other alleles targeting lncRNAs (21 lines), miRNAs (8 lines), CpG islands (13 lines), pseudogenes (3 lines), complexes/clusters/regions (3 lines), multiple protein coding genes (3 lines) or gene segments (2 lines). The raw data for each individual mouse (number of metastases counted) is listed in Table S2. The mutant mice tested were predominantly homozygotes (880 lines, 68%), with heterozygotes generally only being tested (356 lines, 27%) if the line was lethal or sub-viable (*i.e.*, where 0 or ≤13% of homozygote offspring were obtained from heterozygous intercrosses, respectively) and in a small number of cases both heterozygotes and homozygotes were assessed (64 lines, 5%). An IDA was performed on the data from the 256 mouse lines that showed evidence of a phenotype in initial screening and for which at least 3 independent cohorts were tested, and the 34 mutant lines classified as ‘hits’ are shown Table 1.

**Table 1:** Results of the Integrative Data Analysis showing mutant mouse lines with statistically significant decreased or increased pulmonary metastatic colonization (using a statistical threshold of *P* < 0.00055 and a biological threshold of genotype estimate ≤ -55 or ≥ +55). The genotype of the mutant mice listed were all homozygotes except *Id2*, which were heterozygotes. ‘Cohorts’ was the number of individual cohorts that were tested for this particular mouse line. ‘Genotype estimate’ is the alteration in number of pulmonary metastatic colonies for that mutant mouse line relative to control mice. *P* value is the Hochberg test. * The mutant mice (and controls) were administered 1/10^th^ dose of other lines. ** The genotype estimate is an average of the values obtained for both sexes (as there was a sex effect observed); the values for each sex are: 290 ± 26 (male) and 153 ± 31 (female).

GENE	ALLELE	COHORTS	GENOTYPE ESTIMATE	P VALUE
Duoxa2*	*<tm1b(KOMP)Wtsi>*	5	+721 ± 21	2.02E-15
*Irf1*	*<tm1a(EUCOMM)Wtsi>*	4	+246 ± 32	1.12E-04
Rnf10**	*<tm1b(KOMP)Wtsi>*	5	+221 ± 28	1.74E-08
*Pik3cg*	*<tm1a(EUCOMM)Wtsi>*	6	+198 ± 14	7.20E-12
*Herc1*	*<em1Wtsi>*	4	+178 ± 27	2.75E-04
*Arhgap30*	*<tm1a(EUCOMM)Wtsi>*	5	+178 ± 11	2.25E-31
*Dph6*	*<tm1a(KOMP)Wtsi>*	5	+141 ± 13	7.94E-17
*Slc9a3r2*	*<tm1a(EUCOMM)Wtsi>*	6	+141 ± 20	2.11E-05
*Igh-6*	*<tm1(Cgn)>*	6	+101 ± 13	6.78E-08
*Abhd17a*	*<tm1a(KOMP)Wtsi>*	6	+96 ± 11	1.99E-08
*Irf7*	*<tm1(KOMP)Wtsi>*	6	+92 ± 16	5.39E-05
*Id2*	*<tm2b(EUCOMM)Wtsi>*	6	+89 ± 11	4.91E-12
*Fgd4*	*<tm1a(EUCOMM)Wtsi>*	4	+89 ± 15	1.15E-03
*Ankhd1*	*<tm1b(KOMP)Wtsi>*	5	+60 ± 10	1.68E-06
*Fzd6*	*<tm2a(EUCOMM)Wtsi>*	6	+59 ± 10	7.70E-07
*Grsf1*	*<tm1b(EUCOMM)Wtsi>*	7	−55 ± 5	6.36E-12
*Ncf4*	*<tm2Pth>*	8	−61 ± 9	3.84E-08
*Mier1*	*<tm1a(EUCOMM)Wtsi>*	3	−63 ± 13	5.02E-04
*Cybc1*	*<tm1a(KOMP)Wtsi>*	11	−67 ± 11	1.79E-06
*Abraxas2*	*<tm1a(EUCOMM)Wtsi>*	3	−67 ± 11	4.42E-04
*Rwdd1*	*<tm1b(KOMP)Wtsi>*	9	−70 ± 8	1.79E-14
*Cybb*	*<tm1Din>*	6	−71 ± 8	2.76E-12
*Bach2*	*<tm1a(EUCOMM)Wtsi>*	6	−71 ± 7	7.12E-10
*Ncf1*	*<m1J>*	3	−74 ± 10	8.51E-06
*Ncf2*	*<tm1a(EUCOMM)Wtsi>*	6	−77 ± 6	5.99E-13
*Lrig1*	*<tm1a(EUCOMM)Wtsi>*	5	−92 ± 10	2.53E-13
*Cyba*	*<tm1a(EUCOMM)Wtsi>*	6	−99 ± 6	1.18E-17
*Arhgef1*	*<tm1a(EUCOMM)Wtsi>*	6	−100 ± 6	7.03E-14
*Fbxo7*	*<tm1a(EUCOMM)Wtsi>*	4	−100 ± 15	7.63E-08
*Tbc1d22a*	*<tm1b(KOMP)Wtsi>*	6	−102 ± 9	5.12E-22
*Hsp90aa1*	*<tm1(KOMP)Wtsi>*	3	−103 ± 13	6.37E-06
*Entpd1*	*<tm1a(EUCOMM)Wtsi/Hmgu>*	4	−106 ± 16	9.13E-08
*Nbeal2*	*<tm1a(EUCOMM)Wtsi>*	4	−124 ± 14	9.79E-09
*Spns2*	*<tm1a(KOMP)Wtsi>*	10	−160 ± 6	2.81E-37

## Discussion

We have characterized the mechanism of action for several genes that showed a decreased metastatic colonization phenotype in our screen, specifically *Spns2* (van der Weyden *et al.* 2017a), *Nbeal2* (Guerrero *et al.* 2014), *Cybc1* (Thomas *et al.* 2017) and the 5 members of the NOX2 complex (van der Weyden *et al.* 2018). These genes regulate pulmonary metastatic colonization primarily by impacting on the function of the hematopoietic/immune system (lymphocytes, granulocytes/monocytes and platelets). In addition, *Bach2* was also a ‘hit’ in our screen, showing decreased metastatic colonization, and Bach2 is a key regulator of CD4^+^ T-cell differentiation (Roychoudhuri *et al.* 2013), with *Bach2* mutant mice recently being shown to have increased CD8^+^ T-cell cytotoxic activity (Abeler-Dörner *et al.* 2020). Indeed, phenotypic analysis of the 34 metastatic colonization regulating genes detailed in this study show a strong enrichment for genes involved in immune/hematopoietic system development and function with phenotypes in those categories representing 88% of all the reported phenotypes associated with those genes.

As we have previously predominantly focused our attention genes positively regulating metastatic colonization (*i.e.*, mutant mice showing decreased metastasis), we will now turn our focus to discussing negative regulators of metastatic colonization (*i.e.*, mutant mice showing increased metastasis).

### Duoxa2

The strongest biological phenotype we observed, in terms of increased numbers of pulmonary metastatic colonies relative to controls, was with *Duoxa2* mutant mice. The *Dual oxidase maturation factor 2 (DUOXA2*) gene encodes an endoplasmic reticulum protein that is necessary for the maturation and cellular localization (transport from the endoplasmic reticulum to the plasma membrane) of dual oxidase 2 (DUOX2) (Grasberger and Refetoff 2006). The NADPH oxidases, DUOX1 and DUOX2, are critical for the production of extracellular hydrogen peroxide that is required for thyroperoxidase-mediated thyroid hormone synthesis in the thyroid gland; as a result, mutations in DUOX2 and/or DUOXA2 result in thyroid dyshormonogenesis and congenital hypothyroidism (De Deken and Miot 2019). Indeed, *Duoxa2* mutant mice were significantly smaller than their wildtype or heterozygous littermates. A smaller body size (and thus total blood volume) undoubtedly contributed to the increased pulmonary metastatic burden we observed, as well as the presence of extrapulmonary metastases (bone marrow, liver, kidney). However, it was recently shown that *Duoxa2* mutant mice have alterations in key immune cell subsets (CD4+ T-cells, neutrophils, monocytes and NK cells) (Abeler-Dörner *et al.* 2020), which could also account for their increased metastatic colonization. Thus, generation of an inducible *Duoxa2* mutant mouse, wherein *Duoxa2* could be deleted in an adult mouse, will be required to disentangle any effects that loss of DUOXA2 may be having on metastatic colonization aside from a smaller body size.

### Rnf10

The *Ring finger protein 10 (Rnf10*) gene encodes a protein with a ring finger motif (a C3HC4-type zinc finger). *Rnf10*/RNF10 has been shown to be important for key neurobiology functions, including myelin formation (Hoshikawa *et al.* 2008), neuronal cell differentiation (Malik *et al.* 2013) and synaptonuclear messaging (Carrano *et al.* 2019). It has also been reported to play a role in vascular restenosis (Li *et al.* 2019) and a SNP in *RNF10* has been associated with adiposity and type 2 diabetes (Huang *et al.* 2014). We found that *Rnf10*-deficient mice showed increased pulmonary metastatic colonization, with males having a consistently higher metastatic burden than females (290 ± 26 *vs.* 153 ± 31, respectively); this is the only mutant line in which we observed a sexually dimorphic effect. Further investigations are required to provide mechanistic insight as to how *Rnf10* may be regulating metastatic colonization, and why it has a stronger effect in males.

### Slc9a3r2

The *Slc9a3r2* (*SLC9A3 regulator 2*) gene encodes a member of the Na(+)/H(+) exchanger regulatory factor (NHERF) family of PDZ scaffolding proteins. All NHERF proteins are involved with anchoring membrane proteins that contain PDZ recognition motifs to form multiprotein signaling complexes. SLC9A3R2 (also known as NHERF2) has been shown to form complexes with a diverse range of proteins depending on tissue context, including complexing with the lysophosphatidic acid (LPA) receptor and the epithelial anion channel, cystic fibrosis transmembrane conductance regulator (CFTR) in airway and gut epithelia (Zhang *et al.* 2017), the P2Y1 nucleotide and mGluR5 glutamate receptors to different ion channels in neurons (Filippov *et al.* 2010) and megalin and ClC-5 in proximal tubule cells (Hryciw *et al.* 2012). However, it is not yet clear how loss of *Slc9a3r* results in increased metastatic colonization.

### Ankhd1 and Fzd6

The *Ankhd1* (ANKHD1 ankyrin repeat and KH domain containing 1) gene encodes a protein with multiple ankyrin repeat domains and a single KH-domain. ANKHD1 is the mammalian homolog of *Mask1* in Drosophila (which is required for the activity of the Hippo pathway effector, Yorkie) and promotes YAP1 activation and cell cycle progression (Machado-Neto *et al.* 2014). Studies have demonstrated a role for ANKHD1 in promoting cell cycle progression/proliferation in renal, multiple myeloma and prostate cancer cells (Dhyani *et al.* 2012; Machado-Neto *et al.* 2014; Fragiadaki and Zeidler 2018) and in promoting hepatocellular carcinoma metastasis (Zhou *et al.* 2019). The *Fzd6* (frizzled class receptor 6) gene is a member of the ‘frizzled’ gene family, which encode 7-transmembrane domain proteins that are receptors for Wnt signaling proteins. FZD6 has a known role in non-canonical WNT/PCP signaling in cancer (Corda and Sala 2017), including mediating transformation, increased invasiveness of tumor cells and metastasis (Cantilena *et al.* 2011; Corda and Sala 2017; Corda *et al.* 2017). In agreement with this, increased expression of FZD6 has been reported in many cancer types, and correlates with poor prognosis in patients with breast, brain and esophageal cancer (Corda *et al.* 2017; Huang *et al.* 2016; Zhang *et al.* 2019). Thus, both *ANKHD1* and *FZD6* have well-characterized tumor cell-intrinsic roles, however, how they mediate their role in regulating tumor cell- extrinsic metastasis is not clear.

### Regulation of the immune system

Five genes with known roles in regulating immune cell function were identified; the immune cell types included NK cells, lymphocytes (T- and B-cells) and granulocytes/macrophages, which have all been shown to play critical roles in regulating metastasis (reviewed in (Blomberg *et al.* 2018)). Thus genes that interfere with their production, maturation and/or function could understandably result in increased levels of metastatic colonization.

#### Irf1:

The *Interferon regulatory factor 1 (IRF1)* gene encodes a transcription factor that is one of 9 members of the interferon regulatory transcription factor (IRF) family. IRF1 stimulates both innate and acquired immune responses by regulating target genes through binding to an interferon-stimulated response element (ISRE) in their promoters and inducing either transcriptional activation or repression (Ikushima *et al.* 2013). *Irf1* null mice are immunodeficient, characterized by a marked reduction in CD8^+^ T cells (Penninger *et al.* 1997) and a decrease in NK cell numbers with associated impaired cytolytic activity (Taki *et al.* 1997).

#### Irf7:

The *Interferon regulatory factor 7 (IRF7)* gene is another member of the IRF family and plays a critical role in the innate immune response against viruses. *Irf7*-null mice are highly susceptible to H1N1 infection (Wilk *et al.* 2015) and secrete decreased levels of IFN-α/β in response to stimulation (Honda *et al.* 2005).

#### Id2:

The *ID2* (*inhibitor of DNA binding 2*) gene encodes a helix-loop-helix-containing protein that lacks a DNA-binding domain and is one of the four members of the ID family (ID1–ID4). ID proteins dimerize with E protein, RB and Ets transcription factors, preventing the formation of DNA-binding transcription complexes. *Id2* null mice show a greatly reduced population of natural killer (NK) cells, as *Id2* plays a role in NK cell maturation (Yokota *et al.* 1999; Boos *et al.* 2007).

#### Igh-6:

The *IGH-6 (immunoglobulin heavy constant mu)* gene encodes a protein that is important for the production of the heavy chain of IgM antibodies and maturation of pre-B cells, the precursors of B-lymphocytes. *Igh-6* null mice are B-cell-deficient, with their development arrested at the stage of pre-B-cell maturation (Kitamura *et al.* 1991). *Igh-6* null mice also show impaired Th1 T-cell responses to *Salmonella* antigens/infections (Mastroeni *et al.* 2000; Ugrinovic *et al.* 2003) demonstrating a role for B cells in the establishment and/or persistence of a stable T-cell memory pool.

#### Pik3cg:

The *PIK3CG* (*phosphatidylinositol-4,5-bisphosphate 3-kinase catalytic subunit gamma*) gene encodes a class I catalytic subunit of phosphoinositide 3-kinase (PI3K), known by many names, including p110-γ and PI3Kγ. Like other class I catalytic subunits (p110-*α*, p110-*β*, and p110-δ), p110-γ binds a p85 regulatory subunit to form PI3K, which phosphorylate inositol lipids and is involved in the immune response. p110-γ is highly expressed in leukocytes and is important for restraining inflammation and promoting appropriate adaptive immune responses in both humans and mice (Takeda *et al.* 2019). *p110-γ* null mice show defective thymocyte development and T cell activation, as well as neutrophil migration and oxidative burst (Sasaki *et al.* 2000).

### Protein modification

A number of genes encoding protein modifiers were identified; these included a ubiquitin-related protein, a serine hydrolase and a protein involved in amidation. How the targets of these proteins can regulate metastatic colonization is still unclear.

#### Herc1:

The *HERC1* (*HECT and RLD domain containing E3 ubiquitin protein ligase family member 1*) gene encodes an E3 ubquitin ligase protein. In humans, six *HERC* genes have been reported which encode two subgroups of HERC proteins: large (HERC1-2) and small (HERC3-6). The HERC1 protein was the first to be identified and has been found to play numerous roles, including membrane trafficking, protein stability and DNA damage repair, through its interactions with clathrin, TSC2 and pyruvate kinase (M2 isoform), respectively (reviewed in (García-Cano *et al.* 2019)). *Tambaleante* (*tbl*) mice, which carry a spontaneous missense mutation in *Herc1*, show neurological phenotypes including, Purkinje cell degeneration, hind limb clasping and impaired rotarod performance (Mashimo *et al.* 2009). While mutations/loss of *HERC1* expression have been reported in some cancers (reviewed in (García-Cano *et al.* 2019)), it is not clear how tumor cell-extrinsic loss of *Herc1* resulted in increased metastatic colonization.

#### Abhd17a:

The *ABHD17A* (*abhydrolase domain containing 17A*) gene encodes a member of the ABHD17 family of proteins that are membrane-anchored serine hydrolases which can accelerate palmitate turnover on PSD-95 and N-Ras. The catalytic activity of ABHD17 proteins are required for N-Ras depalmitoylation and re-localization to internal cellular membranes (Lin and Conibear 2015) and ABHD17 proteins finely control the amount of synaptic PSD-95 by regulating PSD-95 palmitoylation cycles in neurons (Yokoi *et al.* 2016). More recently, regulation of the palmitoylation status of the transcription factor TEAD, which is depalmitoylased by ABHD17A, has been suggested to be a potential target for controlling TEAD-dependent processes, including cancer cell growth (Kim and Gumbiner 2019).

#### Dph6:

The *Dph6* (*diphthamine biosynthesis 6*) gene encodes a protein that is required for the amidation step of the diphthamide pathway in yeast. Diphthamide is a highly modified histidine residue in eukaryotic translation elongation factor 2 (eEF2) and diphthamide synthesis is required for optimal translational accuracy and cell growth (Uthman *et al.* 2013). In eukaryotes, the formation of diphthamide involves a conserved biosynthetic pathway involving 7 members, DPH1-7 that has been predominantly studied in yeast (reviewed in (Schaffrath *et al.* 2014)). However, they do play an import role in mammalian cells as *Dph1* null mice display multiple developmental defects that parallel Miller-Dieker syndrome (MDS) (Yu *et al.* 2014), associated with deletions on chromosome 17p13.3, *Dph3* null mice are embryonically lethal (Liu *et al.* 2006) and *Dnajc24 (Dph4)* null mice almost always die before birth with the few that do survive showing severe developmental defects reminiscent of *Dph1* null mice (Webb *et al.* 2008). Recently, *Dph6* mutant mice were shown to have an immune phenotype with alterations in many innate and adaptive cell lineages (Abeler-Dörner *et al.* 2020), and it is possible that these may be affecting metastatic colonization. Thus, as very little is known about *ABHD17A* and *DPH6* in the context of cancer, it is difficult to precisely speculate how they may be playing a role in tumor cell extrinsic regulation of metastatic colonization.

### Rho GTPase regulating proteins

Rho GTPases are molecular switches that control a wide variety of signal-transduction pathways, including regulation of the cytoskeleton, migration, and proliferation. Rho GTPases can be regulated by GTPase-activating proteins (GAPs) and Rho GDP/GTP nucleotide exchange factors (Rho GEFs). We identified one of each of these family members.

#### Arhgap30:

The *ARHGAP30* (*Rho GTPase activating protein 30*) gene encodes a Rho GTPase-activating protein, with a role in regulating cell adhesion (Naji *et al.* 2011), as well as suppressing lung cancer cell proliferation, migration and invasion (Mao and Tong 2018). In colorectal cancer (CRC), ARHGAP30 levels correlate with p53 acetylation and functional activation (Wang *et al.* 2014), and ARHGAP30 has been proposed as a prognostic marker for CRC (Wang *et al.* 2014), early-stage pancreatic ductal adenocarcinoma (Liao *et al.* 2017) and lung adenocarcinoma (Li *et al.* 2018).

#### Fgd4:

The *FGD4 (FYVE*, *RhoGEF And PH Domain Containing 4)* gene encodes a GEF specific to the Rho GTPase, CDC42. FDG4, also known as FRABIN, contains an actin filament-binding domain (ABD), an Dbl homology domain (DHD), a cysteine rich-domain (CRD), and two pleckstrin homology domains (PHD), which are involved in binding to the actin and activating CDC42 at that vicinity, resulting in actin cytoskeleton reorganization (allowing for shape changes such as the formation of filopodia and lamellipodia) (Nakanishi and Takai 2008). FGD4 overexpression has been observed in pancreatic neuroendocrine neoplasms (Shahid *et al.* 2019) and expression of FGD4 positively correlates with the aggressive phenotype of prostate cancer (Bossan *et al.* 2018). Mutations in this gene can cause Charcot-Marie-Tooth (CMT) disease type 4H (CMT4H), characterized by heterogeneous hereditary motor and sensory neuropathies as a result of demyelination of peripheral nerves (Delague *et al.* 2007).

Although much is known about these two genes and they have well established roles in tumor cell-intrinsic roles in cancer, it is not clear at this stage how they may be mediating an increased metastatic colonization phenotype.

## Conclusion

In summary, we have used the experimental metastasis assay to screen 1,300 genes/genetic loci to identify novel host/microenvironmental regulators of metastatic colonization. We have identified 34 genes whose loss of expression results in either an increased or decreased ability for mouse melanoma cells to undergo metastatic colonization of the lung following tail vein injection. Some of these genes regulate key pathways in immune cell development or function, however many have only been shown to play a role in tumor cell-intrinsic pathways with no known tumor cell-extrinsic functions reported, thus, we have identified numerous novel regulators of pulmonary metastatic colonization, which could represent potential therapeutic targets.
